# Pre-clinical evaluation of LASSBio-1491: From *in vitro* pharmacokinetic study to *in vivo* leishmanicidal activity

**DOI:** 10.1371/journal.pone.0269447

**Published:** 2022-06-06

**Authors:** Aline Cavalcanti de Queiroz, Gisele Barbosa, Victória Regina Thomaz de Oliveira, Hélio de Mattos Alves, Marina Amaral Alves, Vanessa Carregaro, João Santana da Silva, Eliezer Jesus Barreiro, Magna Suzana Alexandre-Moreira, Lidia Moreira Lima

**Affiliations:** 1 National Institute of Science and Technology for Drugs and Medicines (INCT-INOFAR; http://www.inct-inofar.ccs.ufrj.br/), Laboratory for the Evaluation and Synthesis of Bioactive Substances (LASSBio^®^, http://www.lassbio.icb.ufrj.br), Federal University of Rio de Janeiro (UFRJ), Rio de Janeiro, Rio de Janeiro, Brazil; 2 Laboratory of Pharmacology and Immunity (LaFI), Sector of Physiology and Pharmacology, ICBS, UFAL, Maceió, Alagoas, Brazil; 3 Laboratory of Microbiology, Immunology and Parasitology, Center for Medical Sciences, Campus Arapiraca, Federal University of Alagoas, Arapiraca, Alagoas, Brazil; 4 Department of Biochemistry and Immunology, Faculty of Medicine of Ribeirão Preto, University of São Paulo (USP), Ribeirão Preto, São Paulo, Brazil; Iran University of Medical Sciences, ISLAMIC REPUBLIC OF IRAN

## Abstract

Leishmaniasis is a public health issue. It is among the top five parasitic illnesses worldwide and is one of the most neglected diseases. The current treatment disease includes limitations of toxicity, variable efficacy, high costs and inconvenient doses and treatment schedules. LASSBio-1736 was described as antileishmanial drug-candidate to cutaneous leishmaniasis, displaying plasma stability and with no preliminary signals of hepatic or renal toxicity. In this paper, we described the *in vitro* pharmacokinetic study of LASSBio-1491 (a less lipophilic isostere of LASSBio-1736) and it is *in vitro* and *in vivo* leishmanicidal activities. Our results demonstrated that LASSBio-1491 has high permeability, satisfactory aqueous solubility, long plasma and microsomal half-lives and low *in vitro* systemic clearance, suggesting a pharmacokinetic profile suitable for its use in a single daily dose. The antileishmanial effect of LASSBio-1491 was confirmed *in vitro* and *in vivo*. It exhibited no cytotoxic effect to mammalian cells and displayed good *in –vivo* effect against BALB/c mice infected with Leishmania major LV39 substrain, being 3 times more efficient than glucantime.

## Introduction

Leishmaniasis is one of the most neglected diseases. It is endemic parasitosis in Asia, Africa, Americas and Mediterranean region. Different *Leishmania* species causing various clinical manifestation of leishmaniasis [1, 2]. According to World Health Organization (WHO), in 2018 the most cases of visceral leishmaniasis (VL) has occurred in Brazil, East Africa and India, while over 85% of new cutaneous leishmaniasis (CL) cases has happened in 10 countries (Afghanistan, Algeria, Bolivia, Brazil, Colombia, Iran, Iraq, Pakistan, Syrian Arab Republic and Tunisia) [3].

Despite countless scientific efforts dedicated to prevention and treatment of the Leishmaniasis, the disease remains unmet medical needs. The usual pharmacological treatment is based on few drugs option with substantial efficacy and safety limitations [4–7]. Several authors have dedicated efforts in search for new hits, leads and drug-candidates to treat leishmaniasis based on phenotypic and/or target-based screening [8–10]. Considering the target-based approach, cysteine proteases are emerged as an important target due their key role in the replication, virulence, and survival of protozoa of the Trypanosomatidae family, such as the parasites from the genus *Leishmania* and *Trypanosoma* [11, 12]. Based on a ligand drug design strategy, Alves and coworkers has described a series of putative cysteine protease inhibitors having a new peptidomimetic framework. The authors have identified LASSBio-1736 as antileishmanial drug-candidate for cutaneous leishmaniasis, displaying plasma stability and no preliminary signals of hepatic or renal toxicity [13].

**Fig 1:**
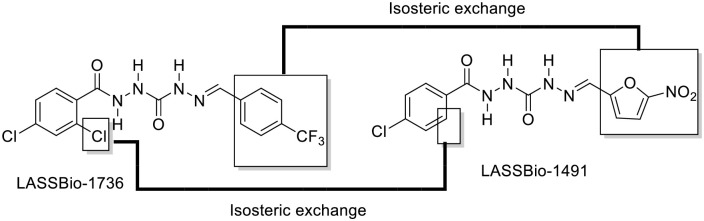
Chemical structure of LASSBio-1491, an isostere of LASSBio-1736 designed by isosteric replacement of monovalent groups (Cl x H; CF_3_ x NO_2_) and ring exchange (phenyl x 2-furanyl).

In this paper, we described the pre-clinical study of LASSBio-1491, a less lipophilic isostere of LASSBio-1736 (Fig 1), bearing a 5-nitrofuran group, a common fragment used to explore antiprotozoal activity [14–17], aiming to compare their *in vitro* pharmacokinetic (PK) and to establish its antileishmanial activity *in vitro* and *in vivo*.

## Materials and methods

### Aqueous solubility

The solubility assay was carried out by correlating the concentration with the absorbance obtained by ultraviolet. First, a stock solution of the compound was prepared by dissolving 1 mg in 20 mL of methanol, and then the wavelength of greatest absorption of each compound was obtained by scanning between wavelengths from 200 nm to 500 nm. From the stock solution, six dilutions were prepared (dilution factor: 2.5), and the absorbance reading was performed in quartz cuvettes (10 mm optical path) in the UV-VIS spectrophotometer (Femto 800XI) at the previously determined wavelength. With the values obtained, a calibration curve with linear regression was made, in which the linearity of the analytical method is observed, with values of correlation coefficient (p > 0.95).

The compound was dissolved in a phosphate buffer solution (pH 7.4), prepared according to the Brazilian Pharmacopoeia (Vol.1) [18] to obtain a supersaturated solution. The supersaturated aqueous solution was kept under stirring for 4 hours at 37°C and then the sample was filtered on a 0.45 μm filter to carry out the reading at the corresponding wavelength of the compound. The solubility was then determined through the equation of the straight line obtained by the linear regression of the calibration curve of each compound [19].

### Parallel Artificial Membrane Permeability Assay (PAMPA)

The parallel artificial membrane permeability assay uses 96-well plate in a “sandwich” type system, where one plate overlaps the other (PAMPA) [20, 21]. The upper plate called the donor compartment refers to the place where the compounds (tests or controls) are diluted in a buffered medium, which is characterized by the presence of a synthetic membrane of PVDF (polyvinylidene fluoride) impregnated with a lipid solution, forming a barrier through which the compounds migrate through a diffusion process to the lower plate called the receptor [20, 22]. The lipid mixture that impregnates the PVDF filter has a different constitution for the permeability tests for the blood-brain barrier (Brain lipid from pig extract in dodecane) and gastro-intestinal tract (*L-α* soy phosphatidylcholine in dodecane). In the PAMPA BBB and TGI assays, the optical density values obtained in the reading at each selected wavelength, for each of the compounds were analyzed in comparison with the values of several controls. In the case of the BBB, these values were used to elaborate an equation and determine the permeability coefficient (*Pe*), using a previously elaborated spreadsheet, and, for the TGI, these values were used to determine the absorbed fraction (Fa%), both using the Excel^®^ program. The permeability result for PAMPA-TGI classifies the compounds according to the percentage of absorbed fraction (Fa%), as: high intestinal permeability (70–100%), medium permeability (30–69%) or low permeability (0–29%), and the samples were diluted from stock solution of 10 mM [23]. The PAMPA-BHE model classifies the compounds only as: permeable (CNS+) or non-permeable (CNS-) and the assay were made from stock solution of 1 mg for each compound [21, 24].

The PAMPA assay was validated using drug was performed using data on artificial membrane permeability of several drugs reported in the literature and compared with results obtained experimentally.

### Plasma stability

For analysis of plasma stability, the LASSBio-1491 in a final concentration of 5 μM from stock solution of 1 mM, was added in 50 μL of rat plasma solution diluted in 250 μL with PBS (pH 7.4) and was placed in a shaker at 37 ºC under vigorous stirring for 0, 30, 60, 120 and 240 minutes. After each reaction time, 500 μL of cold methanol and 500 μL of cold acetonitrile containing 5 uM of methyl 1,1’biphenyl-4-carboxylate 5 μM (internal standard) were added to the wells to stop the reaction. The solution was mixed and centrifuged at 24500 x g for 15 min (Universal centrifuge 320R Hettich^®^). The supernatant (1 mL) was filtrated and placed in vials to be analyzed by HPLC-PDA, Shimadzu—LC20AD, Kromasil C-18 column (4.6 mm x 250 mm, 5 μm), SPD M20A diode array detector, flow 1 mL/min, mobile phase acetonitrile: water 1:1 (v/v) for LASSBio-1491 and acetonitrile: water 60:40 (v/v) for LASSBio-1736, for 20 minutes at 254 nm wavelength [13].

### Microsomal metabolism

The study of the microsomal metabolism of the compounds was evaluated by placing 1 mg/ml rat liver microsome in a microcentrifuge tube, in the presence of a NADPH generator system (1 mM NADP^+^, 1.5 mM MgCl_2_, 3.5 mM glucose-6-phosphate and 0.5 U/mL glucose-6-phosphate dehydrogenase) and phosphate buffer pH 7.4 in sufficient quantity for 250 μL. After 15 minutes of pre-incubation at 37°C in a water bath, the enzymatic reactions were started by adding 2 μL of a stock solution 1 mM from one of the prototypes (500 μL 20% DMSO 80% CH_3_CN), so that a final concentration of 5 μM was reached (0.2% DMSO, 0.8% acetonitrile) [25, 26].

Then, the samples were again incubated at 37°C under agitation for predetermined and fixed periods (0, 15, 30, 45 and 60 minutes). At the end of the incubation period 500 μL of cold methanol and 500 μL of cold acetonitrile containing 5 μM of methyl 1,1’biphenyl-4-carboxylate 5 μM (internal standard) were added to the wells to stop the reaction and the samples were homogenized and kept in an ice bath to accelerate protein precipitation. The mixture was centrifuged at 24500 x g for 15 minutes at 4°C (Universal centrifuge 320R Hettich^®^) [27, 28]. Finally, the supernatant (1 mL) was filtrated and placed in vials to be analyzed by HPLC-PDA, Shimadzu—LC20AD, Kromasil C-18 column (4.6 mm x 250 mm, 5 μm), SPD M20A diode array detector, flow 1 mL/min, mobile phase acetonitrile: water 1:1 (v/v) for LASSBio-1491 and acetonitrile: water 60:40 (v/v) for LASSBio-1736, 20 minutes at 254 nm wavelength. Then, the microsomal half-life and clearance were calculated [26, 27].

### Parasite culture

*L*. *major* promastigotes LV39 (MRHO/SU/59/P) were obtained from João Santana da Silva at Ribeirão Preto Medical School- USP. *L*. *major* IOC/L0581 promastigotes (MHOM/SU/1973/5-ASKH) were obtained from Leishmania collection of the Oswaldo Cruz Institute—Fiocruz. *L*. *major* promastigotes LV39 (MRHO/SU/59/P) and *L*. *major* IOC/L0581 (MHOM/SU/1973/5-ASKH) were grown in a Schneider’s medium (Sigma-Aldrich, St. Louis, MO) supplemented with 500 U/ml of penicillin, 500 mg/ml of streptomycin (Invitrogen e Gibco, Carlsbad, CA, USA), 20% heat-inactivated fetal calf serum (Cultilab, Campinas, SP, Brazil), and 2% sterile male human urine.

### Cytotoxicity against spleen cells

Stock solutions of LASSBio-1491 and pentamidine at the concentration of 100 mM were prepared in DMSO immediately before use. To carry out the suspension preparation of spleen cells, BALB/c mice were euthanized, and an incision was made to remove the spleen. The spleens were macerated using the end of the plungers from a 1 mL syringe in a 70 μm strainer placed in a Petri dish containing RPMI medium. After centrifugation at 1954 x g for 10 minutes at 4 ºC, the supernatant was discarded and 4 mL of red cell lysis solution (82,9 mg of NH_4_Cl, 10 mg of KHCO_3_, 0,372 mg of EDTA in 10 mL of Milli-Q water, using sterilization by filtration) was added to the pellet. After incubation for 4 min at room temperature, the cells were washed with RPMI medium. Subsequently, samples from three animals were pooled and the cell suspension was passed through a 40 μm strainer. Cells were counted in a Neubauer chamber and splenocytes were plated at a concentration of 5 x 10^5^ cells per well in a 96-well culture plate. LASSBio-1491 or pentamidine were added at different concentrations (10^−2^ up to 100 μM). Moreover, spleen cells were also cultured just with medium (free of compounds or vehicle) or with DMSO 0.1%. Then, cells were incubated for 24 h at 37 ºC in an incubator with 5% CO_2_. After, the the propidium iodide incorporation was performed according to the instructions of the Propidium Iodide Kit (Sigma, Copenhagen, Denmark). The splenocytes were analyzed in a FACS Canto II flow cytometer. The data files were saved using FACS Diva software and data analysis was done using Flow Jo software. The cytotoxic activity of pentamidine and LASSBio 1941 was determined by comparing the results obtained in relation to control groups of cells treated only with RPMI medium or vehicle (0.1% DMSO). Data obtained from experiments were expressed as the mean ± standard error of the mean (S.E.M.) of spleen died cells and statistical differences between the treated and the vehicle groups of experiments were evaluated by ANOVA and Dunnett hoc tests.

### *In vitro* activity against promastigote forms of *Leishmania major*

The determination of the leishmanicidal activity of LASSBio-1491 against *L*. *major* promastigotes was carried out according to the methodology described by Rangel and colleagues (1996) [28]. Stationary phase *L*. *major* promastigotes harvested after the third passage (obtained from parasites frozen) and cultured in Schneider’s medium containing 2% human urine and 10% FBS, were counted in a Neubauer chamber and plated at a concentration of 1 x 10^5^ parasites per well in a 96-well culture plate. Then, LASSBio-1491 or pentamidine were added to the wells at different concentrations (10^−2^ up to 100 μM). The promastigotes were also cultured just with Schneider’s medium with 2% human urine and 10% FBS (free of compounds or vehicle, being the basal growth control) or with 0.1% DMSO (vehicle control). After 48h of incubation in a Biochemical Oxygen Demand (B.O.D.) incubator, the promastigotes were counted using a hemocytometer [28]. The results obtained were expressed as the mean ± S.E.M. of the number of promastigotes from triplicate cultures of representative assays, with the data analyzed by ANOVA and Dunnett hoc tests, considering a significance level of p < 0.05. In addition, the linear regression analysis from the Kc values at employed concentrations was used to determine the concentration required to give 50% death of cells (IC_50_).

### *In vitro* activity against amastigote forms of *Leishmania major* and nitrite determination

The evaluation of leishmanicidal activity against *L*. *major* amastigotes was performed according to Garcia and colleagues (2010) [29]. Briefly, female BALB/c mice (6 to 8 weeks old) received 1 mL sterile of 3% sodium thioglycolate by intraperitoneal route (i.p.) and were euthanized after 4 days. The intraperitoneal lavage was collected from the peritoneal cavity of mice using sterile PBS and cells were counted in a hemocytometer [30]. Peritoneal adherent cells (macrophages) were plated at a concentration of 2 x 10^5^ cells per well in 24-well culture plates containing glass coverslips. Subsequently, *L*. *major* promastigotes (harvested after the third passage) were added at a concentration of 2 x 10^6^ parasites per well, aiming to infect the macrophages. After 4 hours, the wells were washed with PBS and treated or not with LASSBio-1491 or pentamidine at different concentrations (100, 50, 25, 10 and 1 μM). After 24 hours in an incubator at 37 ºC and 5% CO_2_, the coverslips were washed with PBS and stained with Giemsa-May Grünwald. The number of amastigotes in 100 macrophages was determined by counting using optical microscope (100x objective). Furthermore, the cell culture supernatant was also collected for nitrite measurement using the Griess reaction [31], an indirect way of determining the concentration of nitric oxide in the supernatant of infected macrophages from the experimental groups. The results obtained were expressed as the mean ± S.E.M. of the number of amastigotes or nitrite concentration from duplicate cultures of representative assays, with the data analyzed by ANOVA and Dunnett hoc tests, considering a significance level of p < 0.05. In addition, the linear regression analysis from the Kc values at employed concentrations was used to determine the IC_50_.

### *In vivo* activity against *Leishmania major* LV39 substrain

All experiments in this work were approved by the Ethics Committee for Animal Experimentation of the Federal University of Alagoas, Brazil (protocol number 2013.02). To alleviate the suffering of mice, all animals used were manipulated in accordance with standards established by Zimmerman (1983) [32]. Thus, 6-week-old female BALB/c mice weighing approximately 20 g were used. The *in vivo* assay was performed according to Pereira and colleagues (2010) [33]. Briefly, ketamine (80mg/kg) and xylazine (8 mg/kg) were simultaneously used to anesthetize the mice by intraperitoneal injection. The promastigotes of *L*. *major* LV39 substrain in the stationary phase, harvested after the third passage (obtained from parasites frozen), were inoculated subcutaneously at a concentration of 1x 10^5^ parasites in the right ear of mice. After three weeks (period necessary to establish the infection in mice), the animals were treated with LASSBio-1491 at a dose of 10 μmol/kg x 28 days (i.p.) or glucantime (meglumine antimoniate) at 30 μmol/kg x 28 days (i.p.). The dose of LASSBio was 3 times lower than that of glucantime, because we wanted to determine whether the test substance at a lower dose was more active than the standard drug. The size of the lesions was measured using a pachymeter. Thiopental (150 mg/kg, i.p.) was used to euthanize the mice and the quantitative limiting-dilution assay was used to determine the parasite burden of infected ears and draining lymph nodes [34]. The results obtained from experiments were expressed as the mean ± S.E.M. with the data analyzed (size of the lesions or parasite burden) by ANOVA and Dunnett hoc tests, considering a significance level of p < 0.05.

## Results

### *In vitro* pharmacokinetic studies

The comparative aqueous solubility of LASSBio-1491 and its isostere LASSBio-1736 was experimentally determined by ultraviolet spectroscopy in a buffer solution of pH 7.4 at 37 ºC. As expected, LASSBio-1491 exhibited higher aqueous solubility (8.72 μM) than LASSBio-1736 (0.26 μM).

The permeability potential of LASSBio-1491 was investigated by *in vitro* parallel artificial membrane permeability assay (PAMPA), using two different membranes to simulate blood-brain barrier (BBB) and gastrointestinal tract (GIT). These assays were performed using two to five wavelengths per compound, in triplicate and in two different analyses (n = 2). The results were expressed as mean (obtained from the mean of the two runs (n = 2) ± standard deviation (SD)). The validation of the methodology was carried out using experimental controls (several known drugs), which were used in the construction of a standard curve (S1–S7 Figs). As demonstrated in Table 1, LASSBio-1491 displayed a BBB permeability of 2.96 x 10^−6^ cm s^-1^, while LASSBio-1736 showed permeability of 10.63 x 10^−6^ cm s^-1^. The permeability across PAMPA-GIT was also determined. LASSBio-1491 exhibited GIT permeability of 4.08 x 10^−6^ cm s^-1^ with a percentage of absorbed fraction of 78.8% (Table 2). Its isostere, LASSBio-1736, was insoluble in the experimental set. Therefore, its GIT permeability could not be determined.

**Table 1:** Permeability coefficient of standard drugs, LASSBio-1491 and LASSBio-1736 determined by PAMPA-BBB assay.

Compounds	*Pe* literature (10^−6^ cm s^-1^)	*Pe* experimental (10^−6^ cm s^-1^)	Classification	cLog P
**Atenolol**	0.8	0.46	CNS -	-
**Caffeine**	1.3	0.81	CNS -	-
**Diazepam**	16	14.30	CNS +	-
**Enoxacin**	0.9	0.53	CNS -	-
**Ofloxacin**	0.8	1.29	CNS -	-
**Testosterone**	17	16.85	CNS +	-
**Verapamil**	16	15.40	CNS +	-
**LASSBio-1491**	-	2.96	CNS+/-	2.14
**LASSBio-1736**	-	10,63	CNS+	4,54

**Table 2:** Permeability coefficient of standard drugs and LASSBio-1491 determined by GIT-PAMPA assay.

Compounds	*Pe* literature (10^−6^ cm s^-1^)	*Pe* experimental (10^−6^ cm s^-1^)	Fa literature (%)	Fa experimental (%)	Classification	cLog P
**Acyclovir**	0.06	0.05/	2.1	1.70	Low	-
**Atenolol**	0.1	0.09	5.2	2.26	Low	-
**Ceftriaxone**	0.1	0.10	1	1.37	Low	-
**Coumarin**	22.9	25.23	100	99.99	High	-
**Diclofenac**	12.5	13.01	100	99.28	High	-
**Hydrocortisone**	3.4	3.70	91	96.34	High	-
**Norfloxacin**	0.9	0.70	55	52.19	Medium	-
**Ranitidine**	0.5	0.30	35	38.29	Medium	-
**Sulfasalazine**	0.3	0.50	42	44.66	Medium	
**Verapamil**	9.7	7.08	98	93.22	High	-
**LASSBio-1491**	-	4.08	-	78.80	High	2.14
**LASSBio-1736**	-	*N.D.	*N.D.	*N.D.	*N.D.	4.54

N.D. = not determined.

* Insoluble at the experimental conditions.

The metabolism rate, elimination rate constant (*k*) and half-life of LASSBio-1491 was determined in rat plasma and rat liver microsomes to investigate its metabolic profile in comparison with LASSBio-1736. As depicted in Table 3, LASSBio-1491 showed low metabolic lability in plasma with only 15.11% of metabolic conversion and plasma half-life (*t*_*1/2*_) of 19.25 h; while LASSBio-1736 displayed t_1/2_ 28.9 h.

**Table 3:** Pharmacokinetic parameters determined from the *in vitro* plasma stability studies of LASSBio-1491 and its isostere LASSBio-1736.

Compounds	Metabolism rate (%)	Elimination rate constant (*k*)	t_1/2_ (h)	Recovery rate (%)
**LASSBio-1491**	15.11	0.0006	19.25	88.52
**LASSBio-1736**	7.30	0.0004	28.9	59.89

The metabolic stability of LASSBio-1491 and its isostere LASSBio-1736 was investigated by incubation in rat liver microsomes in the presence and absence of cofactor (i.e., NADPH regenerating system). As depicted in Table 4, both compounds showed great metabolic stability displaying microsomal *t*_*1/2*_ of 2.68 h and 3.5 h, and intrinsic clearance (*Cl*_*int*_) of 1.075 and 0.825 μL/min/mg, respectively.

**Table 4:** Pharmacokinetic parameters determined from the *in vitro* microsomal stability studies of LASSBio-1491 and its isostere LASSBio-1736, using rat liver microsomes in the presence and absence of cofactor.

Compounds	Metabolism rate non co-factor ^a^ (%)	Metabolism rate with co-factor ^a^ (%)	Elimination rate constant ^b^ (*k*)	*t*_*1/2*_ ^b^ (h)	Volume (μL/mg)	*Cl*_*int*_ ^b^ (μL/min/mg)	*Cl*_*app*_ ^b^ (mL/min/Kg)
**LASSBio-1491**	10.87	25.46	0.0043	2.68	250	1.075	0.166
**LASSBio-1736**	5.81	18.33	0.0033	3.50	250	0.825	0.128

^a^ Co-factor: NADPH regenerating system; Animal weights: 385,0 g; Liver weights: 12,0 g; Microsomal protein: 59,77 mg/mL.

^b^ Calculations performed for the assay with co-factor

*Cl intrinsic* (μL/min/mg) = Volume x (0,693/t_1/2_)

*Cl apparent* (mL/min/mg) = (0.693/t_1/2_ min) x (incubation volume mL/microsomal protein mg) x (microsomal protein mg/liver g) x (liver g/rat weight kg).

### *In vitro* cytotoxic and leishmanicidal activities

Cytotoxic profile of LASSBio-1491 and the standard pentamidine was investigated against mammalian cells by iodide propidium stain method, using spleen cells. The cells were treated with the target compounds at 100 μM and compared with cells treated with vehicle (DMSO 0.1%). As depicted in Table 5, after 24 h of incubation compounds did not affect the viability of spleen cells. Pentamidine was used as standard drug. The choice of pentamidine considered its use in the leishmaniasis treatment, and the *in vitro* inactivity of pentavalent antimonials (front-line drugs of choice for all the clinical forms of leishmaniasis) against promastigotes and its less potency in vitro against amastigotes [35–37].

**Table 5:** *In vitro* cytotoxicity effect of pentamidine and LASSBio-1491 against spleen cells and promastigote forms of *L*. *major*.

Substance	Spleen cells		*L*. *major* promastigote 5-ASKH substrain		*L*. *major* promastigote LV39 substrain		*L*. *major* amasitigote 5-ASKH substrain		*L*. *major* amastigote LV39 substrain	
	IC_50_ ^a^ μM	Maximum effect ^b^ (% ± S.E.M.)	IC_50_ ^a^ μM	Maximum effect ^b^ (% ± S.E.M.)	IC_50_ ^a^ μM	Maximum effect^b^ (% ± S.E.M.)	IC_50_ ^a^ μM	Maximum effect ^b^ (% ± S.E.M.)	IC_50_ ^a^ μM	Maximum effect^b^ (% ± S.E.M.)
Pentamidine	>100	-	0.8 ± 0.1	72.6 ± 2.0**	5.7 ± 0.2	88.1 ± 0.1**	17.1 ± 5.6	53.8 ± 1.1**	8.1 ± 0.9	63.2 ± 2.9**
LASSBio-1491	>100	-	7.5 ± 0.3	68.4 ± 0.1**	7.7 ± 0.1	68.3 ± 0.2**	7.3 ± 0.6	67.5 ± 4.8**	8.3± 0.3	60.2 ± 1.9**

^a^ IC_50_ is the concentration required to give 50% death of parasites, calculated by linear regression analysis from the Kc values at employed concentrations (100, 50, 25, 10, and 1 μM). This constant corresponds to the slope resulting from plotting the log of the growth measurement versus time for each drug concentration.

^b^ Maximum effect is expressed as mean ± standard error of maximum toxicity average of triplicates of a representative experiment.

The values of maximum effect were considered significant when *p < 0.05, **p < 0.01 compared to the 0.1% DMSO group.

The *in vitro* evaluation of the anti-promastigote activity of LASSBio-1491 was assessed against *L*. *major* 5-ASKH and LV39 substrains, as demonstrated in Table 5. The standard drug (pentamidine) and LASSBio-1491 were equipotent against *L*. *major* LV39, while LASSBio-1491 was about 10 times less potent against *L*. *major* 5-ASKH. Both compounds displayed similar maximum effects against the two substrains of *L*. *major* (Table 5).

Further, the leishmanicidal activity against intramacrophage amastigotes of *L*. *major* 5-ASKH and LV39 substrains was investigated and the results are depicted in Table 5. LASSBio-1491 was equipotent to pentamidine against *L*. *major* LV39 and it was two times more potent than the standard drug against *L*. *major* 5-ASKH. Both compounds displayed comparable maximum effects (Table 5).

**Fig 2:**
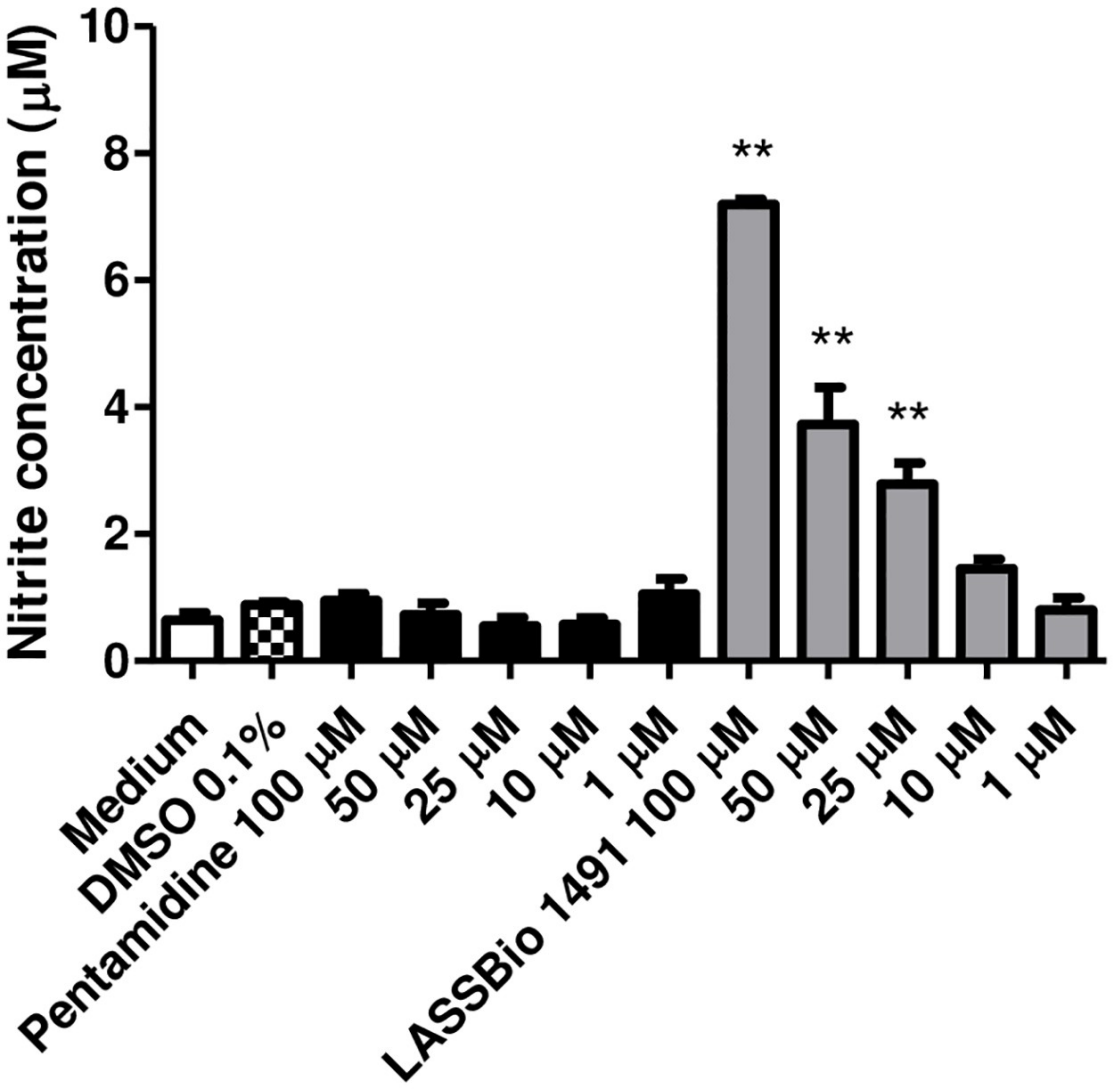
Concentration-dependent response to pentamidine and LASSBio-1491 in the nitrite measurement using the Griess reaction, in culture of peritoneal macrophages infected with *L*. *major* LV39.

It is known that NO has an important microbicidal activity in the macrophage against the parasite. Thus, the involvement of NO production on the cytotoxic activity of LASSBio-1491 against intracellular amastigotes of *L*. *major* was investigated, as shown in Fig 2. LASSBio-1491 increased NO production by BALB/c mice macrophages infected with *L*. *major* in a concentration dependent manner, while pentamidine did not.

### *In vivo* antileishmanial activity

**Fig 3:**
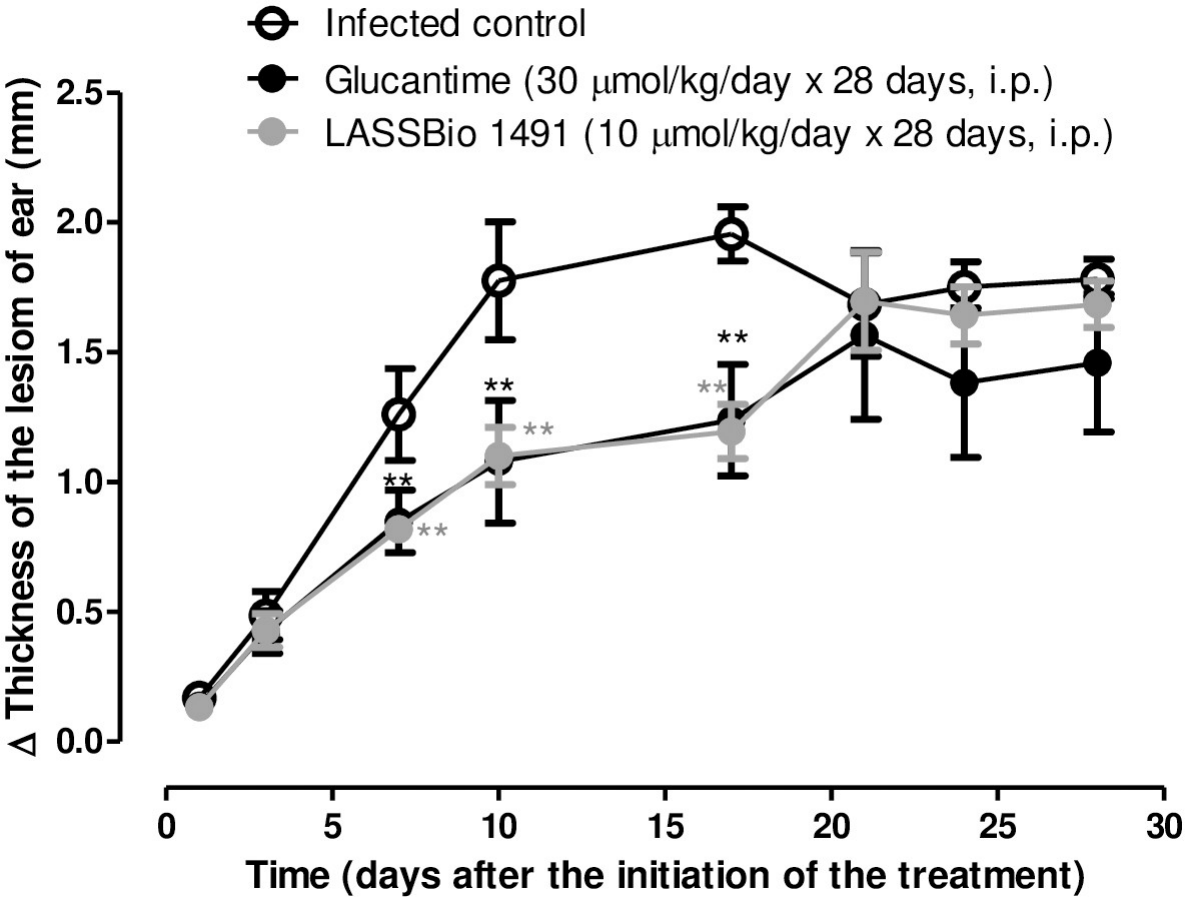
*In vivo* efficacy of LASSBio-1491 (10 μmols/kg/day x 28 days, i.p.) and glucantime (30 μmols/kg/day x 28 days, i.p.) in BALB/c mice infected with *L*. *major* LV39. Lesion sizes were monitored weekly. Values are the mean of lesion sizes in five mice in each group and bars represent the standard error of the mean. The values were considered significant when *p < 0.05, **p < 0.01 compared to the infected control group.

**Fig 4:**
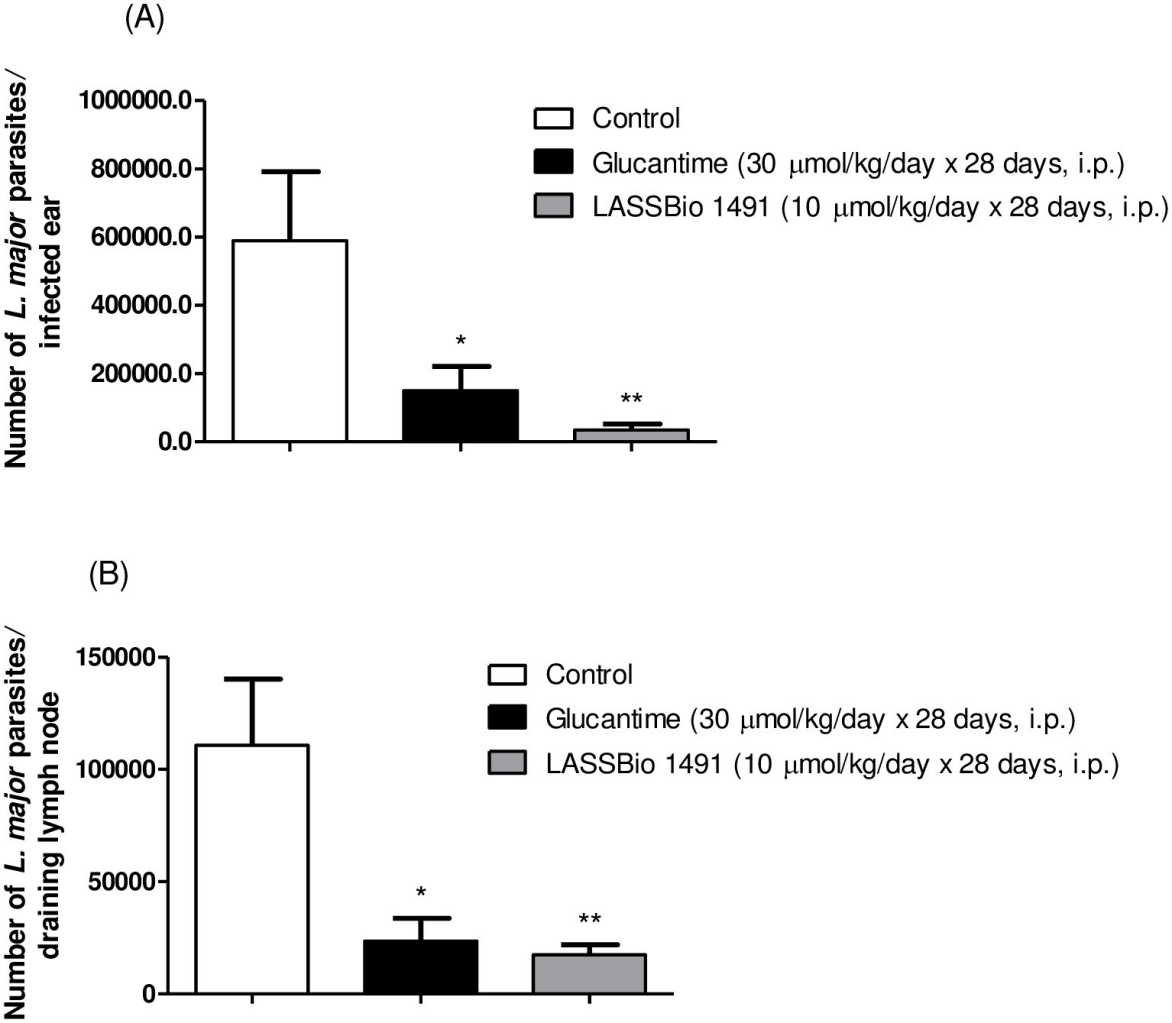
Parasite burden throughout the course of treatments with LASSBio-1491 (10 μmols/kg/day x 28 days, i.p.) and glucantime (30 μmols/kg/day x 28 days, i.p.) in BALB/c mice infected with *L*. *major* LV39. (A) Number of *L*. *major* parasites in the infected ears. (B) Number of *L*. *major* parasites in the draining lymph node. The parasite loads of infected ears and draining lymph nodes were determined using a quantitative limiting-dilution assay. The values represent the mean parasites loads of five mice in each group, and the bars represent the standard error of the mean. *P < 0.05, **P < 0.01 vs. control.

The antileishmanial effect of LASSBio-1491 (10 μmol/Kg/day x 28 days, i.p.) in BALB/c mice infected by ear intradermal inoculation of *L*. *major* LV39 substrain was examined, in comparison to the treatment with glucantime (30 μmol/Kg/day x 28 days, i.p.). Glucantime was used as standard drug since this pentavalent antimony (Sb^V^) compound is still considered the first-line therapy for cutaneous leishmaniasis and has lower toxicity to mice, when compared to pentamidine [35]. The Fig 3 shows the result obtained through the weekly monitoring of lesion size in the experimental groups for 28 days. LASSBio-1491 and glucantime reduced lesion size only between days 7 and 17 after the beginning of the treatment (Fig 3). After this period, both compounds did not significantly decrease the lesion in relation to the control. The effect of glucantime and LASSBio-1941 treatment on the parasite load in the ear and draining lymph nodes of the infected mice was investigated (Fig 4). Interestingly, LASSBio-1491 decreased parasite load in infected ear (Fig 4A) and draining lymph node (Fig 4B), at a dose three times smaller than glucantime.

## Discussion

LASSBio-1491 is a less lipophilic isostere of LASSBio-1736, a compound previously described as a leishmanicidal drug-candidate bearing a new peptidomimetic framework. The improved solubility profile was expected by the replacement of the 4-(trifluoromethyl)phenyl subunit at LASSBio-1736 by a 5-nitrofuran subunit, a classical fragment explored in the designing of antiprotozoal agents (Fig 1). The comparative thermodynamic solubility of LASSBio-1491 and LASSBio-1736 was determined by UV analysis. Aqueous solubility is a critical property in drug discovery and development, being essential to assure reliability of biological assays and to contribute to oral absorption and bioavailability [38]. As expected, LASSBio-1491 exhibited a greater aqueous solubility (8,72 μM or 3,06 mg/mL) than LASSBio-1736 (0,26 μM or 0,109 mg/mL). The comparison between calculated partition coefficient (cLogP) and the experimental aqueous solubility of LASSBio-1491 (cLogP 2.14 and 8,72 μM) and LASSBio-1736 (cLogP 4.54 and 0,26 μM) indicates a better hydrolipidic profile for LASSBio-1491.

The ability of LASSBio-1491 permeates cell membranes was simulated using PAMPA-BBB and PAMPA-GIT assays (Tables 1 and 2). These non-cell-based assays are widely used in early drug discovery process to predict passive, transcellular permeability of hits, leads and drug-candidates [39]. While LASSBio-1736 was predicted as able to cross the blood-brain barrier, LASSBio-1491 displayed a lower PAMPA-BBB and could not be predicted as permeable or impermeable to BBB, showing permeability (Pe) of 2.96 x 10^−6^ cm s^-1^, falling into the method’s blurring range (Table 1). Regarding PAMPA-GIT, LASSBio-1491 was predicted as permeable (4.08 x 10^−6^ cm s^-1^) to gastrointestinal tract, showing a high absorption rate (78.8%, Table 2). The permeability of LASSBio-1736 through PAMPA-GIT assay was not predicted, due to its insolubility under assay conditions. The satisfactory hydrolipidic profile of LASSBio-1491, associated with its permeation across GIT, suggests that this compound may undergo transcellular absorption by passive diffusion.

To obtain data on the metabolic stability and clearance of LASSBio-1491, we determined the *in vitro* half-life (*t*_*1/2*_), the intrinsic (*Cl*_*int*_) and apparent (*Cl*_*app*_) clearances, using rat plasma and rat liver microsomes, in comparison with LASSBio-1736. As shown in Tables 3 and 4, both compounds showed high plasma and microsomal stability. These data agree with the previous information described by Moraes (2017) and Alves (2020), indicating that LASSBio-1736, despite its peptidomimetic nature, has a great stability in rat plasma [13, 17]. The plasma half-life of LASSBio-1736 and LASSBio-1491 was 19.25 h and 28.87 h, respectively. These data suggest that both compounds are not significantly metabolized by plasma hydrolases (Table 3).

These data were confirmed by the metabolism study in rat liver microsomes (RLM) in the absence of NADPH regenerating system. In that condition, LASSBio-1491 and LASSBio-1736 exhibited metabolism rates of only 10.87% and 5.81%, respectively (Table 3). The incubation of both compounds using RLM in the presence of NADPH regenerating system, condition in which oxidative enzymes (CYP450 and FMO) are activated [40], also resulted in a low metabolic rate (25.46% and 18.33%). The microsomal half-life of LASSBio-1491 and LASSBio-1736 was 2.68 h and 3.5 h, respectively (Table 1). The data suggest that both compounds have high stability to oxidative metabolism using RLM, which is consistent with their chemical structures. Consequently, LASSBio-1491 and LASSBio-1736 showed low liver clearance, as can be seen from their *Cl*_*int*_ and *Cl*_*app*_ values (Table 4). The data are important to determinate if a prototype will be able to reach target tissues in leishmaniasis, since liver is an important organ for leishmanicidal activity [41, 42]. Pharmacokinetic characteristics such as long half-life, low systemic clearance and adequate liver penetration are desirable for leishmanicidal drugs, as reported by Moraes & colleagues [17] in *in vivo* studies with LASSBio-1736.

Once established the *in vitro* PK profile of LASSBio-1491, we investigated its antileishmanial effect. In a first approach, we studied the leishmanicidal activity against intramacrophage amastigotes since they are the parasite stage most relevant to human disease. Considering that they reside within an acidic parasitophorous vacuole inside host cells and that the parasite bears a glycoinositolphospholipid coat, limited or impaired uptake of xenobiotics could be observed [43, 44]. The effects of LASSBio-1491 in amastigote forms of *L*. *major* were studied and their intrinsic parasite cytotoxicity was determined (Table 5). This compound showed high anti-amastigote activity against *L*. *major* 5-ASKH substrain with IC_50_ 7.3 ± 0.6 μM and maximum effect (E_max_) of 67.5 ± 4.8%, being 2.3 times more potent than pentamidine. Similar results were found against amastigotes of *L*. *major* LV39, being LASSBio-1491 equipotent to pentamidine with IC_50_ values of 8.3 ± 0.3 μM versus 8.1 ± 0.9 μM, respectively (Table 5).

These results revealed the ability of LASSBio-1491 to exhibit antileishmanial effect against amastigotes of two different *L*. *major* substrains (LV39 and 5-ASKH). In general, LV39 (MRHO/SU/59/P), a southern Russia strain, is more virulent and less susceptible to nitric oxide (NO) than the World Health Organization [3] reference strain 5-ASKH (Turkmenskaya, MHOM/SU/73/5-ASKH) [45, 46]. We have shown that unlike to pentamidine, LASSBio-1491 increased the NO production by BALB/c mice macrophages infected with *L*. *major* LV39 (MRHO/SU/59/P) in a concentration dependent manner (Fig 2). Therefore, we can speculate that besides the direct leishmanicidal activity of LASSBio 1491 (since this compound presented anti-promastigote activity, such as shown in the Table 5), its ability to increase NO concentration in culture of macrophages infected with *L*. *major* LV39, a major host defense mechanism against *Leishmania* spp. [47, 48], can also contribute for its *in vitro* anti-leishmania effect.

The activation of NO synthase 2 (NOS2) elicited by cytokines outcomes the increases in NO production and the consequent killings of promastigote and amastigote forms of *Leishmania spp*. NOS2 mutant mouse were demonstrated to be highly susceptible to *Leishmania* infection [49, 50]. The ability of phagolysosomal amastigotes to decrease NO production in infected macrophages by inhibition of NOS2 is considered one of the possible mechanisms of resistance of *L*. *major* to the macrophages antimicrobial system [51–56].

Although we have demonstrated the capacity of LASSBio-1491 to increase NO concentration in culture of BALB/c mice macrophages infected with *L*. *major* LV39, the mechanism of action involved in this response remains to be investigated.

Considering the promising *in vitro* PK profile of LASSBio-1491 and its *in vitro* leishmanicidal activity, we decided to investigate the anti-*Leishmania* effect of this compound in a murine model of cutaneous leishmaniasis. The BALB/c mice infected with *L*. *major* LV39 were treated during 28 days with LASSBio-1491 at the dose of 10 μmols/kg/day by intraperitoneal administration. The results were compared to control group (vehicle) and glucantime (30 μmols/kg/day x 28 days, i.p.) and are depicted in Figs 3 and 4. Although, LASSBio-1491 and glucantime did not reduce the size of the lesions at the site of cutaneous inoculation of *L*. *major* LV39 on the last day of treatment, it decreased the parasite load in infected ear (Fig 4A) and draining lymph node (Fig 4B), suggesting a systemic antiparasitic effect. Our hypothesis is that glucantime and LASSBio-1491 cannot control the inflammatory process, probably caused by dead parasite antigens, even inducing a reduction in the parasite load in the infected ear.

The glucantime, at the dose of 30 μmols/kg/day for 28 days, was also able to reduce the parasite load in infected ear and draining lymph node similarly to LASSBio-1491 (10 μmols/kg/day for 28 days), since there was no statistically significant difference between these experimental groups. Such effect in a dose three times lower than the one used for glucantime, indicates the promising *in vivo* leishmanicidal profile of LASSBio-1491. It is worth noting that LASSBio-1491 is a peptide mimetic analogue, structurally unrelated to pentamidine and glucantime. For this reason, we expect an action profile with less toxic effects, although this issue still needs to be addressed.

## Conclusion

Taken together, our results revealed LASSBio-1491 as a leishmanicidal agent, whose action seems to depend, at least in part, on its ability to stimulate an increase in NO concentration. Its high permeability, satisfactory aqueous solubility, long plasma and microsomal half-lives and low *in vitro* systemic clearance, ensure to LASSBio-1491 a pharmacokinetic profile suitable for its use in a single daily dose. Its antileishmanial effect was confirmed *in vitro* and *in vivo*. LASSBio-1491 exhibited a significant *in vivo* effect against BALB/c mice infected with *L*. *major* LV39 substrain, being 3 times more efficient than glucantime. The molecular mechanism of action of LASSBio-1491, including an eventual immunomodulatory action, as well as its possible toxicity profile, must be studied and constitute ongoing goals.

## Supporting information

S1 Fig Calibration curve of LASSBio 1491 for the determination of aqueous solubility. (TIF)

S2 Fig Linear correlation between experimental and literature permeability values for blood-brain barrier permeability. Data represent the mean of triplicates in two different analyzes (n = 2).
(TIF)

S3 Fig Linear correlation between the values of absorbed fraction (Fa %) in the literature and experimental in gastrointestinal tract. Data represent the mean of triplicates in two different analyzes (n = 2).
(TIF)

S4 Fig Plasma stability profile of LASSBio-1491. (TIF)

S5 Fig First order rate constant (*k*) for elimination of LASSBio 1491 in plasma stability. (TIF)

S6 Fig Microsomal metabolism profile of LASSBio-1491. (TIF)

S7 Fig First order rate constant (*k*) for elimination of LASSBio 1491 in microsomal metabolism. (TIF)
