# Supplementary figures and images for: Pre-clinical evaluation of LASSBio-1491: From in vitro pharmacokinetic study to in vivo leishmanicidal activity
Source: PLoS One. 2022 Jun 6;17(6):e0269447. doi: 10.1371/journal.pone.0269447 (PMC9170106; doi:10.1371/journal.pone.0269447)

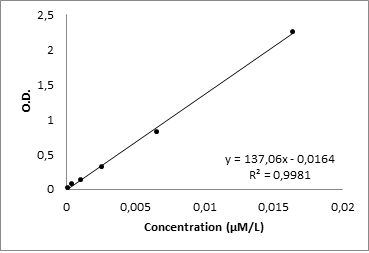

Supplement: S1 Fig — (TIF) [file pone.0269447.s001.tif]

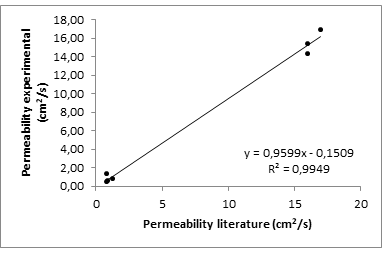

Supplement: S2 Fig — Data represent the mean of triplicates in two different analyzes (n = 2). (TIF) [file pone.0269447.s002.tif]

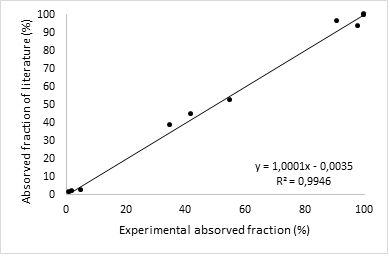

Supplement: S3 Fig — Data represent the mean of triplicates in two different analyzes (n = 2). (TIF) [file pone.0269447.s003.tif]

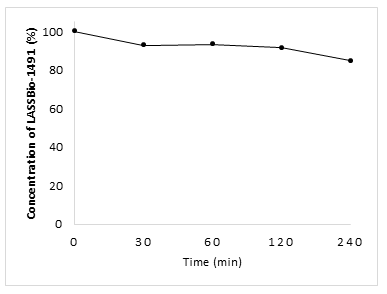

Supplement: S4 Fig — (TIF) [file pone.0269447.s004.tif]

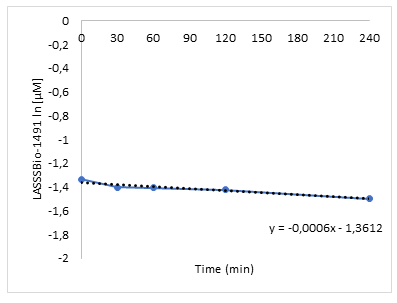

Supplement: S5 Fig — (TIF) [file pone.0269447.s005.tif]

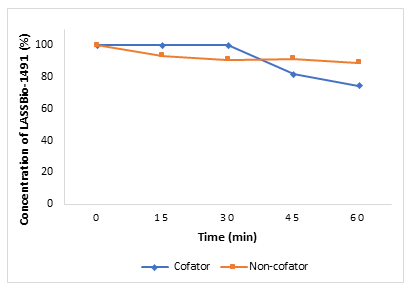

Supplement: S6 Fig — (TIF) [file pone.0269447.s006.tif]

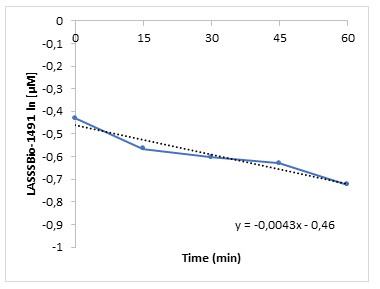

Supplement: S7 Fig — (TIF) [file pone.0269447.s007.tif]
